# Spermidine in dementia: Relation to age and memory performance

**DOI:** 10.1007/s00508-019-01588-7

**Published:** 2019-12-12

**Authors:** Thomas Pekar, Aribert Wendzel, Walter Flak, Alexandra Kremer, Susanne Pauschenwein-Frantsich, Anna Gschaider, Felix Wantke, Reinhart Jarisch

**Affiliations:** 1 Biomedical Science, University of Applied Sciences Wiener Neustadt, Johannes-Gutenberg-Straße 3, 2700 Wiener Neustadt, Austria; 2 Gepflegt Wohnen GmbH, Nierathberg 182, 8412 Allerheiligen bei Wildon, Austria; 3 Federal Office for Viticulture, Gölbeszeile 1, 7000 Eisenstadt, Austria; 4 Privatklinik Rudolfinerhaus GmbH, Billrothstraße 78, 1190 Vienna, Austria; 5 FAZ Floridsdorfer Allergiezentrum, Pius-Parsch-Platz 1/3, 1210 Vienna, Austria; 6 Jarisch&Co GmbH, Währinger Gürtel 45/12, 1180 Vienna, Austria

**Keywords:** Dementia, Alzheimer’s disease, Spermidine, Memory performance, CERAD Plus

## Abstract

Previous studies have highlighted that spermidine has the ability to trigger the important process of dissolving amyloid-beta plaques by autophagy. This manuscript focuses on the correlation of serum spermidine levels between age and between performance in mini-mental state examinations. It will serve as a premise for an ongoing multicentric placebo-controlled study, which focuses on the effect of oral spermidine supplementation on memory performance. Memory tests were carried out on 80 subjects aged 60–96 years old in 6 nursing homes in Styria. Blood samples were taken for the determination of spermidine concentration. The results showed a significant correlation between the spermidine concentration and the mini-mental state examination score (*p* = 0.025). On the basis of the dependence demonstrated it can be concluded that spermidine might be suitable as a biomarker for the diagnosis of neurocognitive changes (senile dementia or Alzheimer’s disease).

## Introduction

Life expectancy is constantly increasing in Austria. Every year of life gained results in a statistically prolonged life expectancy of 3 months. Older people are subject to a decrease in brain performance and memory as they become older. Worldwide 35.6 million people suffer from the consequences of dementia and the number is expected to rise to 115 million by 2050. As a cause for Alzheimer’s disease, neuronal death correlated with aggregated proteins has previously been suspected [1]. The finding of an effective pharmacological treatment for cognitive impairment and the development of biomarkers are top research priorities [1, 2].

Previous studies have highlighted positive effects including lifespan extension by oral spermidine intake [3, 4]. Experiments with the fruit fly *Drosophila melanogaster* showed a cessation of senile dementia and also the restoration of memory performance when spermidine was administered [5, 6]. Spermidine has the ability to trigger the important process of dissolving amyloid-beta plaques by autophagy. Fruit flies supplemented with spermidine showed significant improvements in short and medium-term memory compared to flies of the same age that did not receive the polyamine [6]. Spermidine is contained in semen and especially in red wine [7, 8]. The highest concentration in food was found in wheat germ [9].

The aim of the present study was to investigate whether the serum spermidine level correlates with the memory performance in older individuals. The memory performance was tested by the test battery of the Consortium to Establish a Registry for Alzheimer’s Disease (CERAD), which has been positively evaluated for the early detection of Alzheimer’s disease [10].

## Material and methods

### Participants

A total of 92 older individuals (60–96 years old, fluent German speakers) participated in this study. Participants were recruited through the directors of nursing in six rest homes of the “Gepflegt Wohnen” (Cultivated living) group in Styria, Austria. Persons were eligible for participation in this study if their age was between 60 and 100 years. Furthermore, they had to take part in both the CERAD Plus test and venepuncture. They also had to continue their previous medication. Exclusion criteria included changing previous medication, withdrawal by choice or participation in another study. Written informed consent was obtained from all participants in accordance with the ethics committee of the medical university of Graz. The study was approved by the ethics committee of the medical university of Graz. (30–280 ex 17/18) For patients with advanced dementia, written informed consent was obtained from the legally authorized representatives. In a previous unpublished investigation, the serum spermidine levels in 74 individuals (5–81 years old) were measured in order to get normal values for different age groups. Written informed consent for blood examination of biogenic amines was obtained from the participants or their parents.

### Study design

This article presents provisional results of a randomized, placebo-controlled, double blind multicentric longitudinal study, which has the aim of determining whether the administration of spermidine in wheat germ can improve the results of memory tests.

### Memory test

Memory performance was measured using the CERAD Plus test battery [11]. The CERAD test battery provides tests of verbal fluency (by naming animals), an abbreviated version of the Boston naming test (15 items), mini-mental state examination (MMSE), word list learning/recall/recognition test, constructional praxis, and recall of constructional practice. In CERAD-plus, the trail making test A (TMT-A), trail making test B (TMT-B) and a phonematic fluency test (“s-words”) are included.

### Laboratory procedure

Blood was drawn by venepuncture and collected into serum tubes (Greiner, Kremsmünster, Austria). After centrifugation at 2000*g the serum was stored at −80 °C for further analysis. Serum levels of spermidine in participants were determined by a commercially available enzyme-linked immunosorbent assay (ELISA, abx585001; Abbexa, Cambridge, UK) according to the manufacturer’s instructions. For calculating the sample concentration four standards (3.13–200 pg/ml) were used. Intraassay and interassay variability are <10% and <12%. Measurements were performed by a biomedical scientist who was blinded to all data.

### Statistical analysis

Statistical analyses were carried out with the SPSS 25 statistical package (PASW, SPSS; IBM, Armonk, NY). The relationship was analyzed using Pearson correlation and Kendall rank correlation coefficient tau‑b. The Mann-Whitney U‑test was applied for two-group comparison. The level of significance was set at a *p*-value of less than 0.05.

## Results

**Fig. 1:**
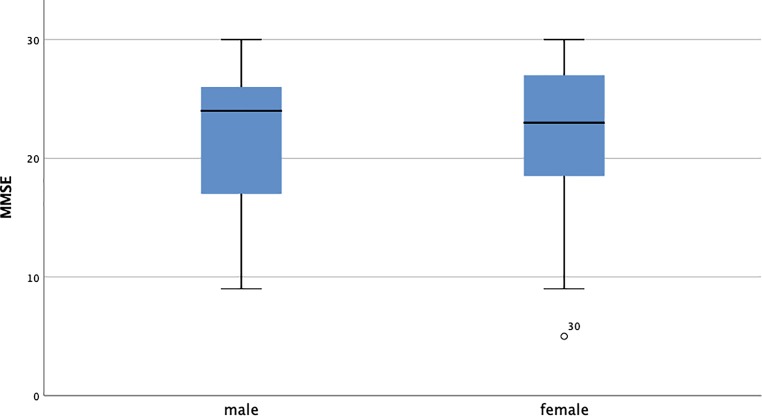
Mini-mental state examination (MMSE) scores in male and female participants. Plots display the median (solid line), interquartile range (box), 10th and 90th percentiles (whiskers)

A total of 80 participants fulfilled the inclusion criteria. The mean age was 83.29 years (interquartile range, IQR 79–90). The distribution between the sexes was 19/61 (male/female). All participants were able to finish the MMSE but 38 participants terminated the questionnaire before all parts of the CERAD Plus had been finished. Therefore, only the results of the MMSE were considered for further examinations. Blood samples were collected within 2 days of the MMSE in 47 (58.8%) and between 3 and 7 days in 33 (41.2%) participants. No statistically significant differences between male and female serum spermidine levels were found (male: mean 47.76 ng/ml, IQR 33–56.75 ng/ml; female: mean 49.19 ng/ml, IQR 38.4–48.19 ng/ml; *p* = 0.545). The mean result of the MMSE was 22.04 (male: mean 24, IQR 17–26.5; female: mean 23, IQR 18.5–27). The difference between male and female MMSE showed no statistically significant difference (male: mean 21.68, IQR 17–27; female: mean 22.15, IQR 18.5–27; *p* = 0.838) (Fig. 1).

**Fig. 2:**
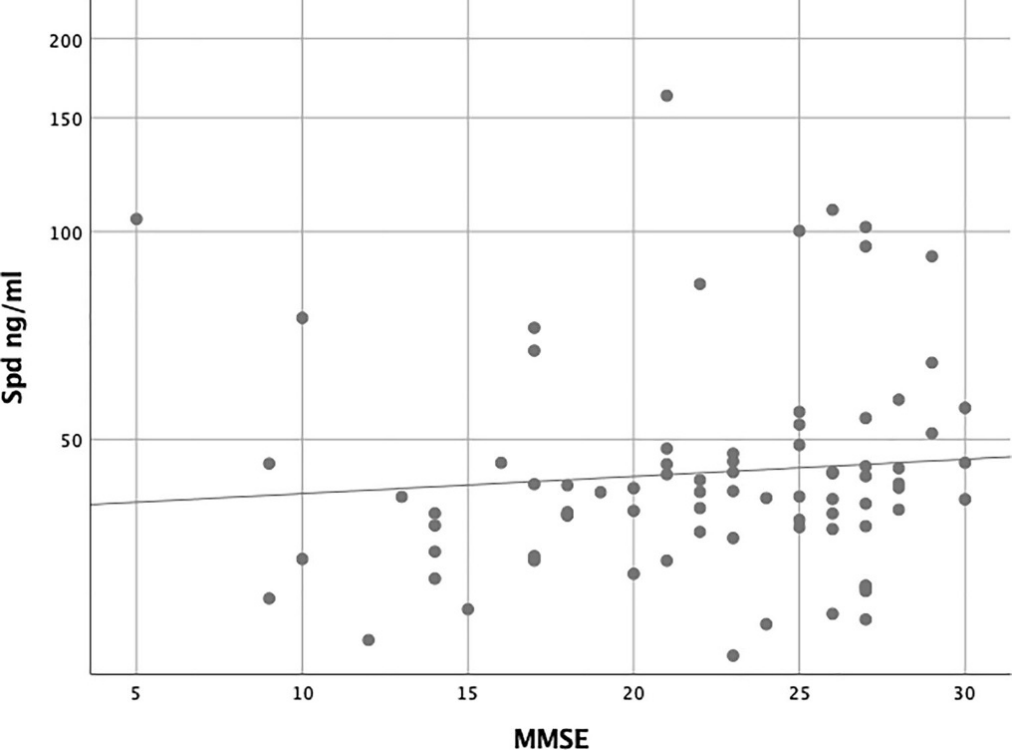
Bivariate correlation between serum spermidine (Spd) levels and mini-mental state examination (MMSE). Kendall rank correlation coefficient tau‑b between spermidine and mini-mental state examination (MMSE) is 0.153 (*p* = 0.025)

The bivariate correlation analyses revealed a significant correlation between the MMSE and the serum spermidine levels (*p* = 0.025) (Fig. 2).

**Fig. 3:**
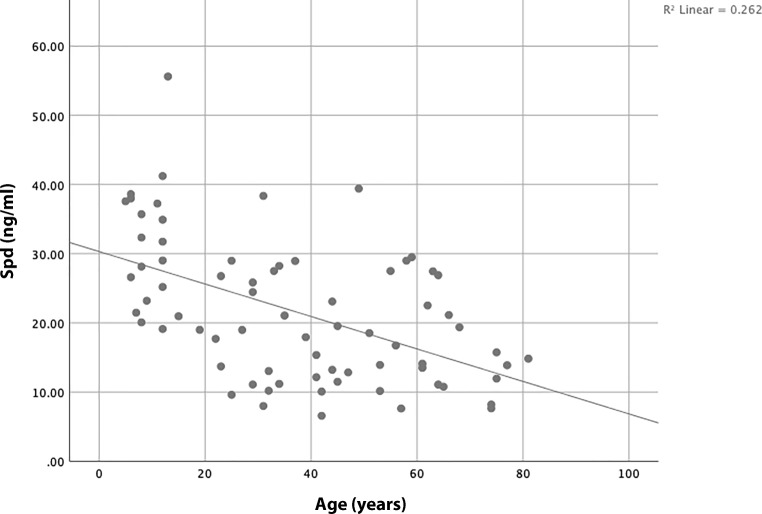
Bivariate correlation between serum spermidine levels and age. Pearson correlation coefficient between spermidine level and age is −0.512 (*p* < 0.001)

The serum spermidine concentrations of 73 healthy controls were measured in a recent pilot study. The mean age was 37.10 years (IQR 14–56.5). The distribution between the sexes was 24/49 (m/f). No statistically significant differences between male and female serum spermidine levels was found (male: mean 22.19 ng/ml, IQR 14–29 ng/ml; female: mean 21.26 ng/ml, IQR 12–27 ng/ml; *p* = 0.481). The bivariate correlation analyses revealed a highly significant negative correlation between serum spermidine levels and age (*p* < 0.001) (Fig. 3).

## Discussion

The present study shows a significant correlation between the spermidine levels in serum and memory performance. Furthermore, the examination of the control group demonstrates that the spermidine level decreases with increasing age. This confirms the assumption that spermidine plays a major role in the occurrence of senile dementia.

Cognitive disorders can have many causes. Circulatory disorders due to atherosclerosis, disorders of thyroid function, changes in vitamin B 12 and folic acid levels may influence cognitive performance [12, 13]. These various possible disorders are also reflected in an even greater scattering of spermidine levels [14]. Nevertheless, there is a significant correlation between spermidine and the memory test values and thus supports the fact that spermidine plays a dominant role in cognitive performance. These findings open up the possibility for routine cerebral examinations of persons over the age of 60 years to detect the onset of cognitive disorders. Validated peripheral biomarkers for Alzheimer’s disease (AD) diagnosis are not available at present [15]. Further work needs to be done to evaluate the qualification of spermidine as a biomarker. Perhaps this will improve the diagnosis of neurocognitive disorders in future, which currently requires a series of examinations including medical history, neuropsychological assessment, and various radiological investigations [16].

The positive effect of spermidine in memory performance in fruit flies has already been proven [5]. Schwarz et al. demonstrated that spermidine supplementation is safe and well-tolerated in mice and older adults [17]. Therefore, a subsequent randomized, placebo-controlled, double blind multicentric longitudinal study is planned, which focuses on the determination of whether the oral administration of spermidine can improve the results of memory tests. The results will be published later this year.
